# Plasma Short-Chain Fatty Acids and Cytokine Profiles in Chronic Kidney Disease: A Potential Pathophysiological Link

**DOI:** 10.3390/ijms27010550

**Published:** 2026-01-05

**Authors:** Anna V. Sokolova, Dmitrii O. Dragunov, Grigory P. Arutyunov

**Affiliations:** Department of Propaedeutics of Internal Diseases No. 1, Institute of Clinical Medicine, Federal State Autonomous Educational Institution of Higher Education ‘N.I. Pirogov Russian National Research Medical University’ of the Ministry of Health of the Russian Federation, 117513 Moscow, Russia; sokolova2211@gmail.com (A.V.S.); arut@ossn.ru (G.P.A.)

**Keywords:** sarcopenia, chronic kidney disease, chronic heart failure, short-chain fatty acids, inflammation, bioimpedance

## Abstract

Sarcopenia is highly prevalent among patients with chronic kidney disease (CKD) and chronic heart failure (CHF), yet the underlying immunometabolic mechanisms remain insufficiently understood. Short-chain fatty acids (SCFAs), inflammatory cytokines, and body-composition alterations may jointly contribute to the development of muscle dysfunction in this population. In this cross-sectional study, 80 patients with CKD and CHF underwent comprehensive clinical, biochemical, bioimpedance, inflammatory, and SCFA profiling. Sarcopenia was diagnosed according to EWGSOP2 criteria. Multivariable logistic regression, LASSO feature selection, correlation analysis, PCA, and Random Forest modeling were used to identify key determinants of sarcopenia. Sarcopenia was present in 39 (49%) participants. Patients with sarcopenia exhibited significantly lower body fat percentage, reduced ASM, and slower gait speed. Hexanoic acid (C6) showed an independent positive association with sarcopenia (OR = 2.24, 95% CI: 1.08–5.37), while IL-8 showed an inverse association with sarcopenia (OR = 0.38, 95% CI: 0.13–0.94), indicating that lower IL-8 levels were more frequently observed in individuals with sarcopenia. Correlation heatmaps revealed distinct SCFA–cytokine coupling patterns depending on sarcopenia status, with stronger pro-inflammatory clustering in C6-associated networks. The final multivariable model integrating SCFAs, cytokines, and body-composition metrics achieved excellent discrimination (AUC = 0.911) and good calibration. Sarcopenia in CKD–CHF patients represents a systemic immunometabolic disorder characterized by altered body composition, chronic inflammation, and dysregulated SCFA signaling. Hexanoic acid (C6) and IL-8 may serve as informative biomarkers of muscle decline. These findings support the use of multidimensional assessment and highlight potential targets for personalized nutritional, microbiota-modulating, and rehabilitative interventions.

## 1. Introduction

Sarcopenia, defined as the progressive loss of skeletal muscle mass, strength, and functional capacity, represents a major medical and social challenge, particularly among older adults and individuals with chronic diseases. In patients with chronic kidney disease (CKD), declining renal function is frequently accompanied by deterioration in muscle status, reduced physical performance, and impaired quality of life [1].

CKD affects millions of individuals worldwide and is associated with a wide spectrum of complications, including metabolic disturbances, nutrient deficiencies, chronic inflammation, and reduced exercise tolerance. These factors collectively contribute to the development and progression of muscle dysfunction in this population [2,3]. Growing evidence indicates that sarcopenia is not only highly prevalent among patients with CKD but also clinically meaningful due to its association with adverse outcomes.

In a study by A.V. Sokolova et al. [4], the presence of sarcopenia in patients with CKD was associated with a greater frequency of renal disease progression (40.5%; *p* = 0.043) and a trend toward elevated IL-6 and IL-18 levels alongside reduced IL-10. Notably, IL-18 demonstrated the strongest prognostic significance, increasing the risk of cardiovascular events by 5.76-fold. Similarly, in a Russian cohort of patients with combined CKD and chronic heart failure (CHF), sarcopenia was identified in 38% and presarcopenia in an additional 39%, indicating that the coexistence of renal and cardiac dysfunction substantially heightens the risk of muscle loss [5].

Parallel to these findings, interest has grown in the potential involvement of short-chain fatty acids (SCFAs) in CKD pathophysiology. Although the association between SCFAs and kidney disease is increasingly recognized, the mechanisms through which these microbial metabolites influence renal function, inflammation, and muscle health remain insufficiently understood. Recent studies highlight the potential therapeutic value of modulating SCFA production through diet or targeted manipulation of the gut microbiota; however, their specific role in the context of sarcopenia and systemic inflammation in CKD requires further investigation [6].

Given these considerations, the primary objective of this study was to examine the relationships between SCFAs, inflammatory markers, and body composition parameters in patients with CKD and CHF, and to determine their potential contribution to the development of sarcopenia. By integrating metabolic, immunological, and bioimpedance-derived measures, this work aims to provide new insights into the mechanisms underlying muscle dysfunction in this high-risk population.

## 2. Results

### 2.1. Study Population Characteristics

A total of 80 patients with chronic kidney disease (CKD) and comorbid chronic heart failure (CHF) were included in the analysis. Among them, 39 patients were diagnosed with sarcopenia, while 41 were classified as non-sarcopenic according to the EWGSOP2 criteria. The demographic, clinical, biochemical, functional, inflammatory, echocardiographic, and body-composition characteristics of the cohort are summarized in Table 1.

The two groups did not differ significantly in age, sex distribution, CKD stage, NYHA functional class, or the prevalence of hypertension, which was present in all participants (*p* > 0.05). Cardiac phenotypes of heart failure were also similarly distributed across groups (*p* > 0.05).

Patients with sarcopenia demonstrated a markedly compromised functional and nutritional profile. Gait speed was significantly slower (0.80 vs. 0.90 m/s, *p* = 0.006), and measures of muscle mass and body composition—including appendicular lean mass, ASM, body weight, BMI, and fat mass percentage—were substantially reduced (all *p* < 0.001). Sarcopenic individuals further showed lower body surface area (*p* = 0.005) and decreased reactance on bioimpedance testing (*p* = 0.013).

Renal function indicators (creatinine, urea, eGFR) were comparable between groups (*p* > 0.05). Among short-chain fatty acids, hexanoic acid (C6) showed a trend toward higher levels in sarcopenic patients (*p* = 0.079). Certain inflammatory cytokines (IL-4, IL-18) demonstrated numerical differences but did not reach statistical significance.

Echocardiographic measurements showed no major structural differences, although LVEF tended to be lower in sarcopenic patients (41% vs. 47%, *p* = 0.064), aligning with established associations between CHF severity and skeletal muscle impairment. NT-proBNP levels were elevated in both groups, reflecting the heart failure burden, but showed no intergroup differences.

Patients with more advanced CKD demonstrated expected reductions in eGFR and increases in creatinine and urea levels (*p* < 0.001). Functional decline was reflected by lower gait speed and SPPB scores in CKD stage 4, although differences did not reach statistical significance. Body composition also varied across CKD categories: patients with CKD stage 2 had higher body weight and BMI, whereas appendicular muscle mass and ASMI were significantly reduced in CKD stage 3a–3b (*p* = 0.036 and *p* = 0.004, respectively). Among inflammatory markers, IL-8 showed a trend toward variation across CKD groups (*p* = 0.078), while SCFA concentrations did not demonstrate significant stage-related differences (Table A1).

Patients with reduced LV function were younger but demonstrated better gait speed and higher handgrip strength compared with those classified in the grey zone, who exhibited the most pronounced functional impairment (*p* < 0.001 for gait speed; *p* = 0.002–0.008 for handgrip strength). Markers of muscle mass, including skeletal muscle mass, ASMI, lean mass, calf and mid-arm circumferences, were substantially higher in the reduced-function group and lowest in the grey-zone group (all *p* < 0.01). Echocardiographic parameters showed expected trends, with markedly lower LVEF and larger LV volumes in the reduced-function phenotype (*p* < 0.001). Inflammatory markers showed selective differences, with IL-6 varying across groups (*p* = 0.041), whereas SCFA concentrations demonstrated no significant variation between phenotypes (Table A2).

### 2.2. Feature Selection Using LASSO Regression

**Figure 1:**
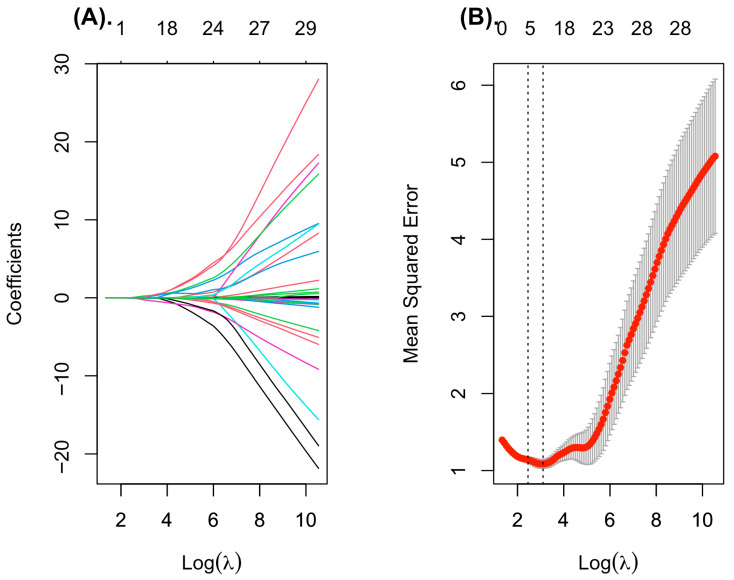
Feature selection using LASSO regression. (**A**) Coefficient profiles for all variables over the sequence of log-transformed lambda values. Increasing regularization leads to coefficient shrinkage, retaining only the most informative predictors. (**B**) Tenfold cross-validation plot illustrating the binomial deviance across log (lambda). Grey bars represent the standard error for each lambda. Vertical dotted lines indicate λ_min and λ_1se; the λ_min value was used to select the final predictors for the multivariable logistic model.

LASSO regression was applied to the study dataset (*n* = 80) to identify the most relevant predictors of sarcopenia in patients with chronic kidney disease. Using tenfold cross-validation, the optimal regularization parameter (λ_min) was selected, and variables with non-zero coefficients at this penalty level were retained for further evaluation (Figure 1).

The final set of predictors selected by the LASSO model included indicators reflecting body composition, cardiac function, and inflammatory/metabolic status. Specifically, the model retained male sex, body weight, fat mass, and left ventricular ejection fraction. Among biochemical variables, standardized hexanoic acid (SCFA) levels, and log-transformed cytokine concentrations (IL-4, IL-8, and IL-18) demonstrated non-zero coefficients and were thus considered relevant contributors. These variables were subsequently included in the multivariable logistic regression analysis to assess their independent association with sarcopenia.

### 2.3. Univariate and Multivariate Logistic Regression Analysis

Univariate logistic regression was first performed to evaluate the individual association of demographic, functional, inflammatory, and metabolic variables with the presence of sarcopenia (Table 2). In the unadjusted analysis, several predictors demonstrated significant relationships with the outcome. Male sex was associated with a higher likelihood of sarcopenia (OR = 2.52, 95% CI: 1.03–6.34, *p* = 0.045). Lower body weight (OR = 0.95, 95% CI: 0.92–0.98, *p* < 0.001) and reduced body fat percentage (OR = 0.87, 95% CI: 0.80–0.92, *p* < 0.001) were both strongly associated with increased odds of sarcopenia. A similar direction was observed for left ventricular ejection fraction, which showed a trend toward significance (OR = 0.97, *p* = 0.068). Among biochemical markers, the z-score of hexanoic acid (SCFA-C6) exhibited a borderline association with sarco-penia (OR = 1.52, 95% CI: 0.96–2.53, *p* = 0.086). Inflammatory cytokines demonstrated heteroge-neous associations with sarcopenia. IL-8 showed a nonsignificant inverse association with the outcome (OR = 0.63, *p* = 0.11), indicating that lower IL-8 concentrations tended to be more prevalent among individuals with sarcopenia; however, this pattern did not reach statistical significance and should be interpreted cautiously, whereas IL-4 and IL-18 did not reach statistical significance.

To identify independent predictors, all variables selected through the LASSO procedure were included in the multivariate logistic regression model. After adjustment for covariates, three predictors remained statistically significant. Lower body fat percentage was indirectly associated with a higher likelihood of sarcopenia, as indicated by an (OR = 0.87, 95% CI: 0.80–0.92, *p* < 0.001). This suggests that higher fat mass may be protective, whereas reduced adiposity reflects greater vulnerability to muscle loss. Higher levels of hexanoic acid (SCFA-C6, z-score) were independently associated with a greater likelihood of sarcopenia (OR = 2.24, 95% CI: 1.08–5.37, *p* = 0.045). In contrast, higher IL-8 concentrations showed an independent inverse association with sarcopenia (OR = 0.38, 95% CI: 0.13–0.94, *p* = 0.050).

Sex, body weight, left ventricular ejection fraction, IL-4, and IL-18 did not retain statistical significance in the adjusted model but exhibited expected directional trends consistent with the univariate analysis.

Taken together, these findings indicate that alterations in body composition, microbial-derived short-chain fatty acids, and specific inflammatory cytokines are key determinants of sarcopenia in patients with chronic kidney disease. The independent contribution of SCFA-C6 and IL-8 highlights a potential interplay between gut-derived metabolites and systemic inflammation in the pathophysiology of sarcopenia.

### 2.4. ROC and Calibration Assessment

**Figure 2:**
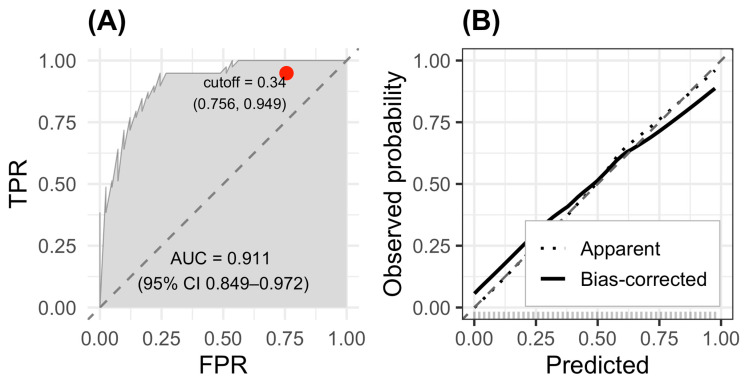
ROC and calibration curves for the sarcopenia prediction model. (**A**) ROC curve showing excellent discrimination (AUC = 0.911; 95% CI 0.849–0.972). (**B**) Calibration plot with 1000 bootstrap resamples.

The predictive performance of the final multivariable logistic regression model was evaluated using receiver operating characteristic (ROC) analysis and calibration assessment (Figure 2). The model demonstrated excellent discriminative ability, yielding an area under the ROC curve (AUC) of 0.911 (95% CI: 0.849–0.972) (Figure 2A). This high AUC value indicates a strong capacity to distinguish between patients with and without sarcopenia. The optimal probability threshold, determined using Youden’s index, was 0.34, corresponding to a sensitivity of 0.756 and a specificity of 0.949.

Calibration of the model was assessed using bootstrap resampling (1000 repetitions). The calibration plot (fig-roc B) showed close agreement between predicted and observed probabilities, with both the apparent and bias-corrected curves closely following the 45-degree ideal line. This alignment indicates that the model is well-calibrated across the full range of predicted risk values and does not exhibit systematic over- or underestimation of sarcopenia probability.

Taken together, the high AUC and favorable calibration results confirm that the proposed model demonstrates robust discriminatory performance and reliable probability estimation, supporting its potential usefulness for clinical decision-making in patients with chronic kidney disease and concurrent heart failure.

### 2.5. Comparison of SCFA–Inflammatory Pathway Correlations by Sarcopenia Status

**Figure 3:**
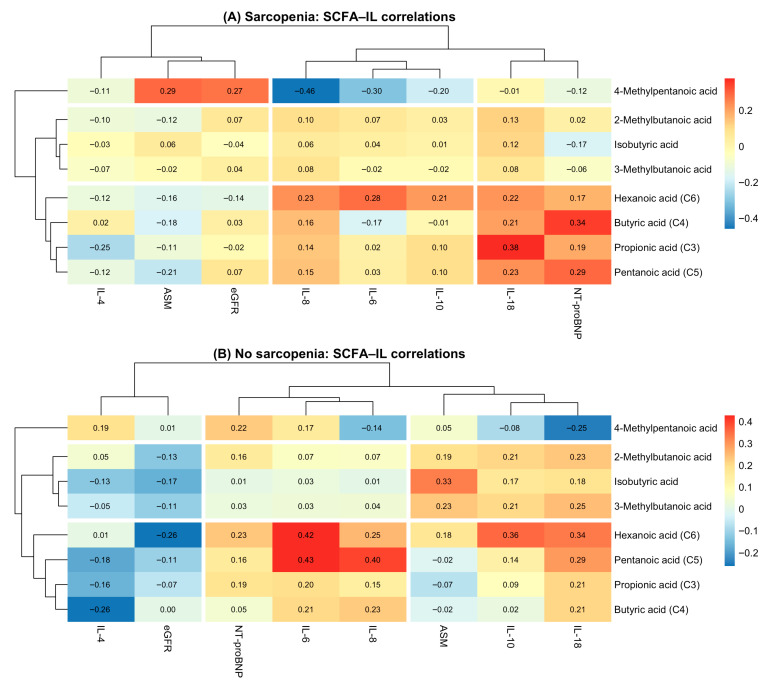
SCFA–cytokine correlation heatmaps in patients with and without sarcopenia. (**A**) Correlation heatmap between SCFAs and inflammatory markers in patients with sarcopenia. (**B**) Correlation heatmap between SCFAs and inflammatory markers in patients without sarcopenia.

Figure 3 illustrates clustered heatmaps comparing the correlation structure between short-chain fatty acids (SCFAs) and inflammatory biomarkers in patients with sarcopenia (Panel A) and without sarcopenia (Panel B). Hierarchical clustering revealed distinct correlation patterns between metabolic and inflammatory pathways depending on sarcopenia status.

In the sarcopenia group, SCFAs formed two major clusters: (1) branched-chain SCFAs (isobutyric, 2-methylbutanoic, 3-methylbutanoic, and 4-methylpentanoic acids), which showed predominantly weak or negative correlations with inflammatory cytokines; (2) straight-chain SCFAs (butyric, propionic, pentanoic, and hexanoic acids), which demonstrated moderate positive correlations with IL-6, IL-10, and IL-18. Notably, hexanoic acid (C6) showed the strongest associations with pro-inflammatory markers, particularly IL-18 and NT-proBNP, highlighting its potential relevance in sarcopenia-related inflammation.

In contrast, the non-sarcopenia group demonstrated a more uniform and consistently positive correlation pattern. Straight-chain SCFAs (C3–C6) clustered tightly with IL-6, IL-8, IL-10, and IL-18, forming a coherent “pro-inflammatory metabolic cluster.” Compared with sarcopenic patients, correlations were generally stronger in magnitude, suggesting more preserved metabolic–inflammatory coupling in individuals without muscle loss.

On the biomarker axis, inflammatory cytokines (IL-6, IL-10, IL-18, and IL-8) grouped together in both panels, whereas NT-proBNP and eGFR formed a separate cluster, reflecting their cardiometabolic rather than immunological profiles.

### 2.6. Principal Component Analysis (PCA) and Cluster Analysis

The adequacy of applying principal component analysis (PCA) was assessed using the Kaiser–Meyer–Olkin (KMO) measure and Bartlett’s test of sphericity. The overall KMO value was 0.66, indicating acceptable sampling adequacy for PCA. Bartlett’s test of sphericity was statistically significant (χ^2^ = 5877.5; df = 1035; *p* < 0.001), confirming the presence of a correlation structure suitable for the application of principal component analysis.

**Figure 4:**
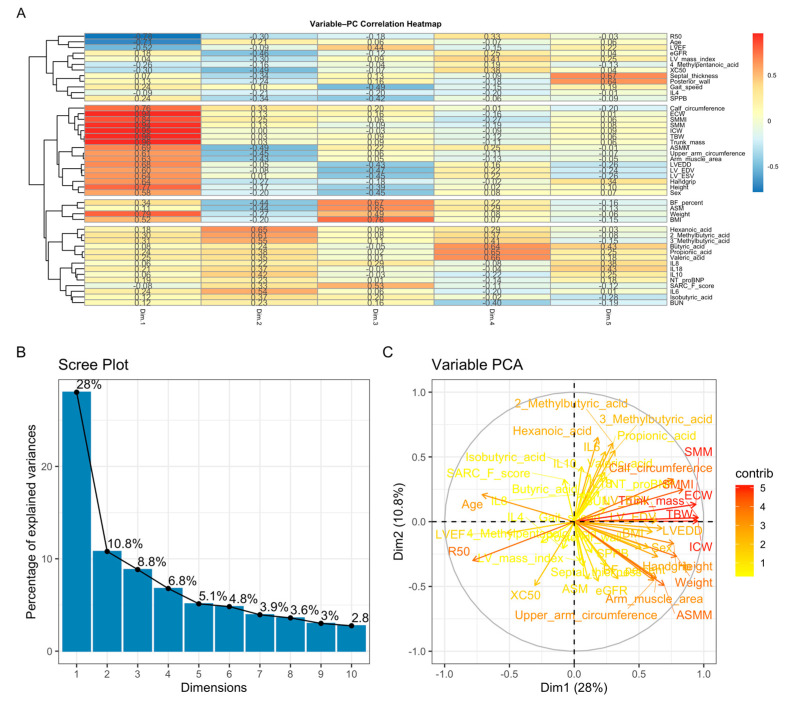
Principal component analysis (PCA). (**A**) Variable–PC correlation heatmap showing clustering of body-composition measures, functional indicators, inflammatory cytokines, and SCFAs according to their loading patterns. (**B**) Scree plot demonstrating that PC1 and PC2 explain 28% and 10.8% of the total variance, respectively. (**C**) PCA biplot illustrating that PC1 primarily reflects body-composition and cardiac structural parameters, whereas PC2 captures functional performance and inflammatory activity; SCFAs display heterogeneous metabolic loadings.

PCA reduced the dataset into 10 components explaining 75.3% of the total variance, with PC1 and PC2 accounting for 28% and 10.8%, respectively. PC1 reflected a body-composition and metabolic axis (SMM, ASM, BMI, BF%, LV mass), whereas PC2 represented a functional–inflammatory axis (gait speed, SPPB, IL-6, IL-8, IL-18, NT-proBNP) (Figure 4). SCFAs contributed variably to both dimensions, forming a separate metabolic cluster. The PCA biplot illustrated these relationships, while the variable–PC correlation heatmap revealed coherent clusters of structural, functional, inflammatory, and SCFA-derived markers.

### 2.7. Random Forest Model

**Figure 5:**
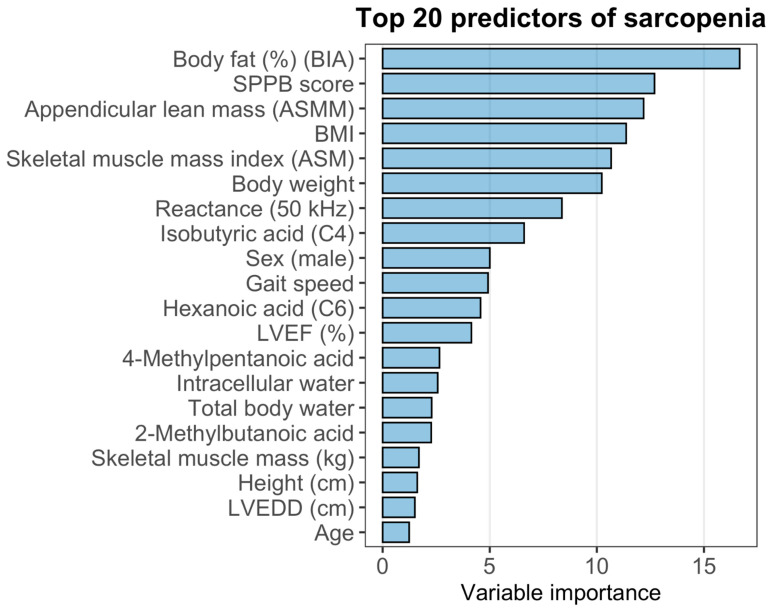
Variable importance and diagnostic performance of the Random Forest model for sarcopenia.

The variable importance ranking derived from the Random Forest model is presented in Figure 5. Among all predictors, body fat (%) measured by bioelectrical impedance had the highest contribution to model performance, followed by SPPB score, appendicular lean mass (ASMM), BMI, and skeletal muscle mass index (ASM). Measures related to body composition (body weight, reactance at 50 kHz, intracellular and total body water) and several short-chain fatty acids (C4, C6, 4-methylpentanoic acid, 2-methylbutanoic acid) also demonstrated substantial importance. Classical clinical predictors such as gait speed, sex, and left ventricular ejection fraction (LVEF) appeared in the mid-range of the ranking, whereas age and LVEDD showed comparatively lower contributions.

To evaluate the discriminative performance of the model, ROC analysis was performed. The Random Forest classifier demonstrated an area under the ROC curve (AUC) of 0.8593, with a 95% confidence interval of 0.7784–0.9401, indicating good discriminatory power for identifying sar-copenia in this cohort.

## 3. Discussion

Our study is among the first to concurrently evaluate SCFAs, inflammatory biomarkers, body composition parameters, and sarcopenia in patients with combined CKD and CHF. This integrated analytical approach provides a more comprehensive understanding of the mechanisms underlying muscle dysfunction in this high-risk population. The findings support the concept that muscle impairment in CKD is a multifactorial process shaped by metabolic disturbances, chronic inflammation, and alterations in body composition. Elevated concentrations of pro-inflammatory cytokines, including tumor necrosis factor-α (TNF-α) and interleukin-6 (IL-6), are known to activate catabolic pathways that promote muscle degradation [7]. In patients with CKD, levels of pro-inflammatory cytokines (IL-1, IL-6, TNF-α, and C-reactive protein) have been shown to inversely correlate with kidney function, thereby perpetuating inflammation and contributing to oxidative stress [6].

At the same time, evidence regarding the interplay between inflammation, gut microbiota, and sarcopenia in CKD remains inconsistent. Although several studies have demonstrated substantial differences in gut microbial composition between patients with and without sarcopenia, these findings were not accompanied by significant differences in circulating uremic toxins (indoxyl sulfate, p-cresyl sulfate) or major inflammatory cytokines (TNF-α, IL-6, IL-17). Moreover, secondary analyses failed to establish a robust association between interleukin-10 levels and gut microbiota composition [8].

In this context, the application of advanced multivariate analytical techniques becomes particularly valuable, as such methods can uncover latent patterns and complex relationships linking microbiota-derived metabolites, inflammatory activity, and clinical phenotypes in CKD-associated sarcopenia [9]. For example, Cheng-Sheng Yu et al. [10] demonstrated that heatmap-based visualization and clustering analyses can successfully identify key biomarkers and delineate patient subgroups with distinct trajectories of CKD progression, including individuals at early stages who exhibit a high risk of rapid decline in renal function.

In our study, the inflammatory profile of patients with CKD and CHF demonstrated heterogeneous associations with cytokines. IL-8 showed an inverse association with sarcopenia in the multivariate model (OR = 0.38, 95% CI: 0.13–0.94), whereas this relationship was not evident in the univariate analysis. This discrepancy likely reflects the complex and dysregulated inflammatory environment characteristic of CKD–CHF, in which chronic immune activation (“inflammaging”) may obscure or modify individual cytokine effects. Importantly, the observed inverse association with IL-8 should not be interpreted as a protective mechanism; rather, it may represent a compensatory response or reflect residual confounding arising from the interplay of multiple inflammatory pathways. Therefore, IL-8 in this context appears to function more as a marker of immunometabolic heterogeneity than as a direct mediator of sarcopenia.

A growing body of evidence supports the notion that the gut microbiota plays a pivotal role in the pathogenesis of CKD through the production of SCFAs—metabolites known to attenuate renal injury by modulating immune and inflammatory responses [11,12]. While the biosynthetic pathways of acetate, propionate, and butyrate are well characterized and widely distributed among numerous bacterial taxa, the mechanisms underlying the production of hexanoic (caproic) acid remain less understood, although recent studies have begun to clarify these pathways [13].

SCFAs are traditionally regarded as metabolites with potent anti-inflammatory properties. They suppress inflammatory reactions by reducing immune cell migration, proliferation, and recruitment, lowering the production of pro-inflammatory cytokines, and inducing apoptosis [14]. SCFAs can enter immune cells through passive diffusion or via specific transport proteins (MCT1/4, SMCT1/2), modulating intracellular signaling pathways and thereby exerting immunoregulatory effects [15].

Systemic and local inflammation can alter the transport of SCFAs, particularly hexanoic acid, into the systemic circulation. Increased intestinal permeability—commonly referred to as “leaky gut”—facilitates the translocation of microbial metabolites into the bloodstream. Under physiological conditions, the colonic epithelium tightly regulates SCFA absorption, with a substantial proportion being utilized by colonocytes to maintain barrier integrity [16].

In the study by Sokolova et al. [17], several short-chain fatty acids—C3, C4, C5, and especially C6—demonstrated correlations with pro-inflammatory cytokines IL-18 and IL-8. The strongest association was observed for hexanoic acid (C6). This may reflect the influence of chronic heart failure activity, as C6 concentrations negatively correlated with muscle mass parameters and estimated glomerular filtration rate. These findings are consistent with our previous results, in which hexanoic acid showed a negative prognostic association in patients with CHF and CKD [18]. Similar results were reported by Sam et al. [19], who demonstrated that C6 exerts pro-inflammatory effects not only by enhancing cytokine production but also by suppressing IL-10 synthesis through activation of the TLR2 signaling pathway.

These findings highlight that while body composition metrics remain the cornerstone of sarcopenia assessment, SCFAs may provide complementary pathophysiological insight rather than serving as standalone biomarkers.

In contrast, other SCFAs—including butyrate, acetate, and propionate—exhibit strong anti-inflammatory and barrier-protective properties. Butyrate activates the GPR109A receptor on epithelial and dendritic cells, modulates the NLRP3 inflammasome, supports physiological IL-18 production, and enhances IL-10 synthesis, contributing to an anti-inflammatory response. Acetate and propionate interact with the GPR43 receptor, promoting the release of PYY and GLP-1, thereby influencing metabolism and intestinal transit. These mechanisms highlight SCFAs as key mediators integrating intestinal barrier function, immune regulation, and systemic metabolic effects, all of which may substantially impact muscle physiology in patients with CKD and CHF [20].

The lack of a significant contribution of age in our predictive models may be explained by the presence of pronounced chronic inflammatory activity in patients with CKD and CHF, which contributes to muscle dysfunction and diminishes the relative effect of chronological aging—a phenomenon consistent with “inflammaging” [21].

Our findings underscore the importance of a comprehensive assessment in patients with CKD and CHF, integrating SCFA profiles, inflammatory markers, and body composition parameters. Such an approach may improve early risk stratification, detection of sarcopenia, and selection of personalized nutritional and rehabilitation strategies.

In this context, it is also important to consider the role of microbiota-derived uremic toxins, as experimental evidence indicates that indoxyl sulfate exerts direct cytotoxic effects on skeletal muscle cells by increasing oxidative stress and apoptosis, thereby contributing to the development of uremic sarcopenia independently of myogenic differentiation pathways [22].

Overall, our results indicate that sarcopenia in CKD and CHF represents a systemic condition shaped by the interplay of metabolic disturbances, inflammation, alterations in body composition, and changes in SCFA profiles. Understanding these mechanisms may facilitate the development of targeted interventions, including nutritional support, modulation of gut microbiota, and structured physical rehabilitation. Further prospective studies are needed to establish causal relationships by assessing the longitudinal dynamics of SCFAs and inflammatory markers and their impact on muscle health.

## 4. Materials and Methods

### 4.1. Study Design and Population

This investigation was designed as an observational, cross-sectional analytical study including patients with chronic kidney disease (CKD) and chronic heart failure (CHF). All clinical, biochemical, inflammatory, short-chain fatty acid (SCFA), and body composition measurements were obtained at a single time point. The study was conducted at City Clinical Hospital No. 4 of the Moscow Healthcare Department (GKB No. 4 DZM) between September 2019 and December 2021 Figure 6. The study was conducted in accordance with the principles of the Declaration of Helsinki and was approved by the Institutional Review Board of Pirogov Russian National Research Medical University (Protocol code No. 137/19, approved on 2 September 2019).

- Inclusion 18 years.
- Confirmed diagnosis of chronic kidney disease (CKD) according to KDIGO guidelines (based on eGFR and/or markers of kidney damage).
- Documented chronic heart failure (CHF) diagnosed in accordance with current clinical recommendations.
- Availability of complete clinical, biochemical, and body composition data, including measurements of: Short-chain fatty acids (SCFAs), nflammatory cytokines, Muscle mass and body composition parameters, Functional performance indicators.
- Ability and willingness to provide informed consent.

Exclusion Criteria:

- Acute Kidney Injury: AKI within the past 3 months
- Active Infections: Current systemic infection or antibiotic use within 4 weeks
- Inflammatory Diseases: Active inflammatory bowel disease, rheumatoid arthritis, systemic lupus erythematosus, or other autoimmune conditions
- Malignancy: Active cancer or cancer treatment within the past 2 years (excluding non-melanoma skin cancer)
- Liver Disease: Chronic liver disease (Child-Pugh class B or C) or elevated liver enzymes >3× upper limit of normal
- Cardiovascular Events: Myocardial infarction, stroke, or major cardiovascular surgery within 3 months
- Gastrointestinal Surgery: Major GI surgery (e.g., bariatric surgery, colectomy) within the past year
- Diabetes (uncontrolled): HbA1c > 10% or diabetic ketoacidosis within 6 months
- Conditions limiting accurate assessment of muscle mass or function, such as limb amputation or severe orthopedic disorders.
- Extreme Diets: Following extreme dietary patterns (e.g., very low carbohydrate, ketogenic) that may significantly alter SCFA production
- Alcohol Abuse: Current alcohol abuse (>14 drinks/week for men, >7 drinks/week for women)
- Substance Abuse: Current illicit drug use

### 4.2. Definition of Sarcopenia

Sarcopenia was diagnosed according to the 2019 European Working Group on Sarcopenia in Older People (EWGSOP2) [23] consensus, which prioritizes low muscle strength as the primary indicator of probable sarcopenia, with confirmation through reduced muscle mass and severity grading based on impaired physical performance. Three components were evaluated: muscle strength, appendicular skeletal muscle mass (ASM), and physical performance.

Muscle strength was assessed using a DK-25 hand dynamometer (Nizhny Tagil, Russia.). Participants performed two maximal grip trials per hand while standing, maintaining the device with the elbow extended. The highest value recorded across all attempts was used for analysis. Low muscle strength was defined as <28 kg in men and <18 kg in women, consistent with EWGSOP2 thresholds [23].

Appendicular skeletal muscle mass (ASMM) Equation (1) was estimated using a validated bioelectrical impedance analysis (BIA) equation (ABC-02, MEDASS, Moscow, Russia). This predictive formula has demonstrated strong agreement with DXA-based measurements in previous validation studies, ensuring accurate and standardized assessment of muscle mass [24].

(1) $$\mathrm{ASMM}=4.211+0.267\cdot \frac{{\mathrm{height}}^{2}}{\mathrm{resistance}}+0.095\cdot \mathrm{weight}+1.909\cdot \mathrm{sex}+0.012\cdot \mathrm{age}+0.058\cdot \mathrm{reactance}$$

Physical performance was evaluated using gait speed. Gait speed was measured over a 4-m walkway at a usual pace; the fastest of two trials was used, with <1 m/s indicating low performance.

Sarcopenia was diagnosed when low muscle strength was accompanied by low muscle mass, and considered severe when reduced physical performance was additionally present.

In accordance with EWGSOP2 recommendations, an SPPB score ≤8 was considered indicative of low physical performance and used to grade the severity of sarcopenia when combined with low muscle strength and reduced muscle mass.

### 4.3. Laboratory Test Indicators

Laboratory test indicators in this study encompassed biochemical markers, renal function parameters, inflammatory cytokines, short-chain fatty acids, and hematological variables relevant to the evaluation of chronic kidney disease and sarcopenia. All laboratory analyses were performed using plasma samples obtained from peripheral venous blood. Renal function was assessed using serum creatinine, blood urea nitrogen (urea), and the estimated glomerular filtration rate (eGFR), calculated using the CKD-EPI creatinine-based equation, which is widely recommended for European-origin populations.

Markers of inflammation included interleukin-4 (IL-4), interleukin-6 (IL-6), interleukin-8 (IL-8), interleukin-10 (IL-10), and interleukin-18 (IL-18), measured using standardized immunoassay methods. N-terminal pro-B-type natriuretic peptide (NT-proBNP) was assessed to evaluate systemic cardiovascular stress.

Short-chain fatty acids (SCFAs) were quantified as individual serum metabolites, including propanoic (C3), isobutyric (iso-C4), butyric (C4), 2-methylbutanoic, 3-methylbutanoic, pentanoic (C5), 4-methylpentanoic, and hexanoic (C6) acids.

Reference values for SCFAs were established based on the monitored multiple reaction monitoring (MRM) transitions of their derivatized forms:

- Propanoic acid (C3): 180.05 → 91.05 ng/mL
- Butanoic acid (C4) and isobutyric acid (iso-C4): 194.10 → 91.05 ng/mL
- Pentanoic acid (C5), 2-methylbutanoic acid (α-C5), and 3-methylbutanoic acid (β-C5): 208.15 → 91.05 ng/mL
- 4-Methylpentanoic acid (iso-C6) and hexanoic acid (C6): 222.10 → 91.05 ng/mL

### 4.4. Definitions of Measurement Cutoffs and Calculations

Body mass index (BMI) categories were defined according to the cutoffs recommended for the adult Russian population and aligned with WHO classification: obesity was defined as BMI ≥ 30 kg/m^2^, overweight as 25–29.9 kg/m^2^, and normal body weight as 18.5–24.9 kg/m^2^.

Estimated glomerular filtration rate (eGFR) was calculated using the CKD-EPI (2009) creatinine-based equation, which is widely used in nephrology practice in Russia and considered suitable for European-origin populations.

Chronic kidney disease (CKD) staging followed the Kidney Disease: Improving Global Outcomes (KDIGO) 2012 guidelines [25], which are standard in the Russian Federation. CKD was categorized into five stages based on eGFR:

- Stage 1: eGFR ≥ 90 mL/min/1.73 m^2^ with markers of kidney damage (e.g., albuminuria);
- Stage 2: eGFR 60–89 mL/min/1.73 m^2^ with markers of kidney damage;
- Stage 3a: eGFR 45–59 mL/min/1.73 m^2^;
- Stage 3b: eGFR 30–44 mL/min/1.73 m^2^;
- Stage 4: eGFR 15–29 mL/min/1.73 m^2^;
- Stage 5: eGFR < 15 mL/min/1.73 m^2^.

Albuminuria categories followed KDIGO [25] recommendations and were defined based on urine albumin-to-creatinine ratio (ACR) as A1 (<30 mg/g), A2 (30–300 mg/g), and A3 (>300 mg/g). Persistent albuminuria was confirmed by at least two abnormal test results obtained over a period of ≥3 months.

### 4.5. Diagnosis of Chronic Heart Failure

Chronic heart failure (CHF) was diagnosed in accordance with contemporary clinical guidelines and required the presence of typical symptoms (exercise intolerance, dyspnea, fatigue, or peripheral edema), objective evidence of cardiac structural and/or functional abnormalities, and reduced or preserved left ventricular ejection fraction confirmed by transthoracic echocardiography. Assessment included measurement of left ventricular ejection fraction (LVEF), chamber dimensions, wall thickness, and diastolic function parameters. In addition, elevated levels of natriuretic peptides (NT-proBNP) were used as supportive diagnostic criteria when available. The diagnosis was established by an experienced cardiologist based on the integrated evaluation of clinical presentation, echocardiographic findings, and laboratory data [26,27].

### 4.6. Statistical Analysis

The statistical analysis was performed using R, version 4.4.2, in the RStudio development environment (packages: ggplot2, ggpubr, dplyr, tidyverse, gtsummary, rstatix, FactoMineR, factoextra, randomForest). The distribution of continuous variables was assessed using the Shapiro–Wilk test, complemented by visual inspection of QQ plots and histograms.

Quantitative data are presented as mean ± standard deviation (SD) for normally distributed variables or as median with interquartile range (IQR) for non-normally distributed variables. Comparisons between two groups were performed using Student’s *t*-test for normally distributed variables or the Wilcoxon rank-sum test for non-normal distributions. For comparisons across more than two groups, ANOVA or the Kruskal–Wallis test was applied, depending on the distribution of the data.

Categorical variables were summarized as frequencies and percentages, and differences between groups were assessed using the Chi-square test or Fisher’s exact test when expected counts were <5. Spearman’s rank correlation coefficient was used to examine associations between continuous and ordinal variables.

Random Forest

A Random Forest classifier was employed to identify the most informative variables among the analyzed biomarkers. This ensemble method constructs numerous decision trees and aggregates their outputs, thereby reducing the instability and overfitting inherent to single-tree models and improving overall classification performance. All analyses were performed using the “randomForest” package in R [28].

Principal Component Analysis and Cluster Analysis

Principal component analysis (PCA) was applied to summarize the variability of clinical, biochemical, inflammatory, and body composition parameters and to address multicollinearity among predictors. All variables except patient identifiers and the sarcopenia outcome were included. The number of retained principal components was selected based on cumulative explained variance, targeting approximately 70–75% of total variance. Hierarchical clustering using Ward’s method was then performed to identify patient groups with similar multidimensional profiles.

Heatmap and Clustering

A heatmap was generated to visualize patterns across clinical and biochemical variables. Hierarchical clustering (Ward’s method) was applied to both participants and variables to identify groups with similar biomarker profiles, which were displayed using color gradients. Heatmap construction and clustering procedures were performed in R using standard visualization packages.

Due to the relatively small sample size and the high dimensionality of candidate predictors, LASSO (Least Absolute Shrinkage and Selection Operator) logistic regression was employed as an exploratory variable selection technique to identify the most informative predictors of sarcopenia while addressing multicollinearity and reducing the risk of overfitting. The variables selected by LASSO were subsequently entered into a parsimonious multivariable logistic regression model, with careful consideration of the events-per-variable rule. Model performance and adequacy were further evaluated using conventional measures of model fit and discrimination, thereby complementing the regularization-based variable selection approach.

All statistical tests were two-tailed, and a *p*-value < 0.05 was considered indicative of statistical significance.

## 5. Conclusions

This study demonstrates that sarcopenia in patients with concomitant CKD and CHF constitutes a systemic condition arising from profound alterations in body composition, persistent low-grade inflammation, and disturbances in microbiota-derived metabolic pathways. Among the evaluated biomarkers, reduced body fat percentage, specific bioimpedance-derived indices, and elevated concentrations of hexanoic acid (C6) emerged as the most robust predictors of sarcopenia, with C6 independently associated with impaired muscle status and heightened pro-inflammatory cytokine activity.

Integrative analysis combining short-chain fatty acid (SCFA) profiles, inflammatory mediators, and detailed body-composition parameters yielded a highly discriminative predictive model, underscoring the central contribution of immunometabolic dysregulation to the pathophysiology of sarcopenia in the CKD–CHF population. These findings highlight the necessity of comprehensive, multidimensional patient assessment and point toward promising avenues for individualized therapeutic strategies, including targeted modulation of the gut microbiota, tailored nutritional interventions, and structured programs of physical rehabilitation.

## 6. Study Limitations

Several limitations should be acknowledged when interpreting the findings of this study. First, the cross-sectional design does not allow causal inferences regarding the relationships between SCFAs, inflammatory markers, body composition parameters, and sarcopenia in patients with CKD and CHF. Second, the sample size was relatively limited, which may reduce statistical power and restrict the generalizability of the results. Third, SCFAs were measured only once in plasma, capturing systemic rather than intestinal production, and their concentrations may be influenced by diet, gut microbiota composition, hepatic metabolism, and concomitant medications. Dietary intake, long-term antibiotic exposure, and fecal SCFA profiles were not collected, limiting the ability to attribute circulating SCFAs to specific microbial or metabolic pathways. Fourth, although bioimpedance analysis is widely used to estimate muscle mass, its accuracy may be affected by fluid imbalance, a frequent condition in CKD and CHF. Finally, residual confounding cannot be excluded, as factors such as physical activity and medication regimens were not systematically evaluated.

## Figures and Tables

**Figure 6:**
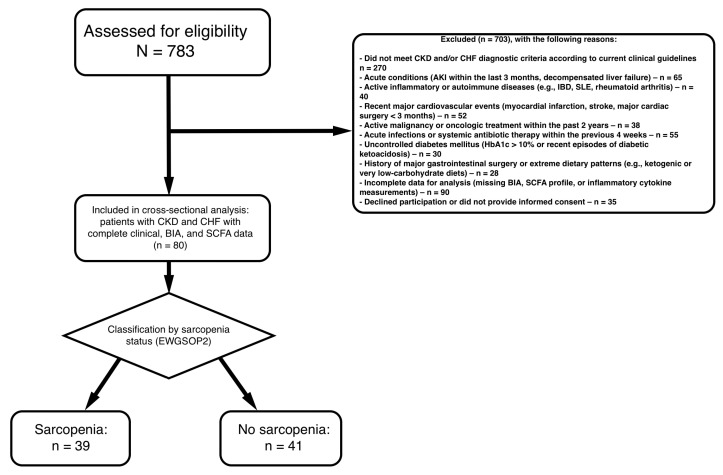
Flowchart of the study population.

**Table 1:** Baseline characteristics stratified by sarcopenia status.

Variable	No *N* = 41 ^1^	Sarcopenia *N* = 39 ^1^	*p*-Value ^2^	Overall *N* = 80 ^1^
Sex			0.071	
Female	24 (59%)	14 (36%)		38 (48%)
Male	17 (41%)	25 (64%)		42 (53%)
NYHA functional class			0.4	
Class I	1 (2.4%)	0 (0%)		1 (1.3%)
Class II	4 (9.8%)	5 (13%)		9 (11%)
Class III	27 (66%)	19 (49%)		46 (58%)
Class IV	2 (4.9%)	3 (7.7%)		5 (6.3%)
None	7 (17%)	12 (31%)		19 (24%)
Hypertension				
yes	41 (100%)	39 (100%)		80 (100%)
Chronic kidney disease stage			0.3	
CKD 2	9 (22%)	5 (13%)		14 (18%)
CKD 3a	10 (24%)	17 (44%)		27 (34%)
CKD 3b	17 (41%)	14 (36%)		31 (39%)
CKD 4	5 (12%)	3 (7.7%)		8 (10%)
HF echocardiographic phenotype			0.2	
HFrEF	16 (39%)	22 (56%)		38 (48%)
HFpEF	15 (37%)	8 (21%)		23 (29%)
HFmrEF	10 (24%)	9 (23%)		19 (24%)
Age at inclusion (years)	68.87 ± 9.55	71.64 ± 10.60	0.2	70.22 ± 10.11
Gait speed (m/s)	0.80 [0.50; 1.25]	0.90 [0.41; 1.50]	0.3	0.80 [0.49; 1.50]
Handgrip strength, left (kg)	28.40 [17.90; 38.50]	31.60 [17.50; 42.05]	>0.9	29.14 [17.60; 40.58]
Handgrip strength, right (kg)	30.30 [23.20; 41.37]	35.40 [20.20; 43.96]	>0.9	32.75 [21.00; 43.15]
Muscle strength index (%)	32.70 [23.11; 44.78]	41.60 [26.27; 53.57]	0.092	38.66 [25.52; 52.07]
SPPB score	7.00 [5.00; 9.00]	6.89 [5.00; 7.15]	0.3	7.00 [5.00; 7.83]
Total body water (L)	46.00 [35.90; 53.80]	46.02 [37.40; 47.90]	0.3	46.01 [36.05; 49.41]
Extracellular water (L)	18.31 [14.70; 21.60]	18.30 [15.00; 19.30]	0.3	18.30 [14.80; 20.00]
Intracellular water (L)	28.20 [21.40; 31.80]	28.10 [22.00; 29.97]	0.6	28.15 [21.55; 30.49]
Fat mass (%)	34.00 [26.47; 40.00]	22.30 [13.20; 26.15]	<0.001	26.22 [19.45; 35.70]
Skeletal muscle mass (kg)	28.26 ± 10.12	28.44 ± 8.08	>0.9	28.35 ± 9.13
Height (cm)	166.42 ± 10.13	166.90 ± 10.53	0.8	166.65 ± 10.27
Skeletal muscle mass index (kg/m^2^)	9.96 [8.30; 11.60]	10.03 [8.20; 11.30]	0.8	10.01 [8.25; 11.35]
Body weight (kg)	92.00 [84.00; 120.00]	83.00 [73.00; 89.71]	<0.001	87.00 [75.90; 98.50]
Body mass index (kg/m^2^)	34.80 [31.05; 39.40]	29.40 [25.70; 32.30]	<0.001	31.90 [28.10; 36.25]
Body surface area (m^2^)	2.03 [1.82; 2.29]	1.94 [1.73; 2.03]	0.005	1.96 [1.76; 2.08]
Lean mass (kg)	62.93 [49.10; 73.40]	62.70 [51.10; 65.60]	0.3	62.92 [49.25; 67.55]
Resistance ( 50 kHz, Ω)	453.72 ± 97.50	448.69 ± 98.40	0.8	451.27 ± 97.35
Reactance (50 kHz, Ω)	48.76 [38.53; 56.09]	41.94 [31.16; 46.73]	0.013	44.92 [35.44; 51.91]
eGFR (mL/min/1.73 m^2^)	45.88 ± 13.45	45.20 ± 11.93	0.8	45.55 ± 12.66
Calf c ircumference (cm)	38.00 [34.81; 41.00]	38.36 [33.97; 41.00]	0.5	38.28 [34.77; 41.00]
Mid-arm muscle c ircumference (cm)	24.19 [23.09; 25.46]	23.58 [22.52; 25.02]	0.061	23.77 [22.79; 25.06]
Mid-arm muscle area (mm^2^)	455.90 [419.73; 504.72]	446.44 [394.74; 491.55]	0.089	452.27 [407.04; 493.76]
Propionic acid (µmol/L)	6310.00 [4560.00; 7145.30]	6400.00 [4640.00; 7243.20]	0.7	6354.35 [4635.00; 7226.60]
Isobutyric acid (µmol/L)	10,200.00 [6710.00; 15,100.00]	11,500.00 [9020.00; 13,040.54]	0.4	10,950.00 [8540.00; 13,456.10]
Butyric acid (µmol/L)	14,500.00 [10,200.00; 18,800.00]	16,100.00 [10,500.00; 19,800.00]	0.4	15,500.00 [10,300.00; 19,153.05]
2-Methylbutyric acid (µmol/L)	8070.00 [3910.00; 10,400.00]	9030.00 [6390.00; 10,700.00]	0.3	8560.00 [4565.00; 10,479.60]
3-Methylbutyric acid (µmol/L)	13,300.00 [7500.00; 18,000.00]	14,400.00 [9850.00; 17,000.00]	0.6	14,000.00 [8880.00; 18,000.00]
Pentanoic acid (µmol/L)	480.00 [371.00; 614.07]	455.00 [324.00; 574.00]	0.6	477.39 [335.00; 605.00]
4-Methylpentanoic acid (µmol/L)	168.62 [118.00; 195.29]	154.00 [136.00; 193.09]	0.3	157.15 [124.00; 194.19]
Hexanoic acid (µmol/L)	1300.00 [792.00; 2049.90]	1870.00 [1200.00; 2399.43]	0.079	1565.00 [1085.00; 2277.81]
NT-proBNP (pg/mL)	2051.00 [1054.34; 3761.25]	2339.79 [1289.01; 3577.78]	0.4	2108.40 [1108.80; 3684.54]
IL-4 (pg/mL)	0.08 [0.00; 0.31]	0.19 [0.00; 0.58]	0.2	0.13 [0.00; 0.40]
IL-6 (pg/mL)	8.20 [4.90; 14.89]	11.35 [6.64; 27.25]	0.3	10.00 [5.56; 18.47]
IL-8 (pg/mL)	28.88 [10.75; 45.44]	14.29 [8.88; 28.47]	0.8	21.11 [9.88; 39.33]
IL-10 (pg/mL)	4.40 [3.20; 7.59]	5.53 [3.20; 8.71]	0.3	4.90 [3.20; 8.00]
IL-18 (pg/mL)	285.62 [181.82; 339.94]	255.19 [213.32; 407.75]	0.3	274.79 [197.08; 403.97]
Creatinine (µmol/L)	123.00 [109.00; 138.00]	125.00 [102.00; 137.00]	0.6	124.19 [106.50; 137.08]
Urea (mmol/L)	7.90 [6.80; 9.45]	8.10 [7.06; 11.06]	0.7	8.04 [7.01; 10.54]
LVEF (%)	47.00 [30.00; 52.00]	38.31 [28.00; 46.00]	0.064	41.18 [30.00; 50.00]
LV mass index (g/m^2^)	106.19 [89.64; 123.37]	98.47 [69.06; 126.00]	0.11	104.89 [82.89; 125.69]
IVS thickness (cm)	1.20 [1.10; 1.25]	1.19 [1.10; 1.21]	0.5	1.20 [1.10; 1.21]
Posterior wall thickness (cm)	1.12 [1.08; 1.20]	1.12 [1.10; 1.20]	0.6	1.12 [1.09; 1.20]
LV EDV (mL)	118.00 [97.00; 155.58]	127.34 [94.90; 165.00]	0.3	125.47 [97.00; 159.09]
LV ESV (mL)	73.61 [50.40; 91.88]	78.00 [53.00; 113.00]	0.4	76.34 [52.00; 99.00]
LV EDD (cm)	5.09 [4.80; 5.70]	5.36 [4.80; 5.70]	0.5	5.31 [4.80; 5.70]
Sex (BIA)	17.00 (41.46%)	25.00 (64.10%)	0.071	42.00 (52.50%)
Appendicular lean mass (kg)	17.82 [16.00; 20.17]	16.79 [14.31; 18.02]	<0.001	16.88 [15.70; 18.86]
Appendicular skeletal muscle mass (kg/m^2^)	6.43 [6.00; 6.79]	5.82 [5.57; 6.14]	<0.001	6.10 [5.73; 6.58]
Maximal handgrip strength (kg)	31.50 [23.60; 42.20]	35.40 [20.20; 46.60]	>0.9	32.90 [21.25; 43.68]

^1^ *n* (%); Mean ± SD; Median [Q1; Q3]. ^2^ Pearson’s Chi-squared test; Welch Two Sample *t*-test.

**Table 2:** Univariate and multivariate logistic regression for sarcopenia predictors.

	Univariate Analysis				Multivariate Analysis		
Predictor	N	OR	95% CI	*p*-Value	OR	95% CI	*p*-Value
Sex (male)	80	2.52	1.03, 6.34	0.045	3.07	0.49, 23.1	0.2
Body weight (kg)	80	0.95	0.92, 0.98	<0.001	0.94	0.87, 1.01	0.13
Body fat percentage (%)	80	0.87	0.80, 0.92	<0.001	0.88	0.77, 0.98	0.034
Left ventricular ejection fraction (LVEF, %)	80	0.97	0.93, 1.00	0.068	0.95	0.89, 1.01	0.10
Hexanoic acid (SCFA-C6), z-score	80	1.52	0.96, 2.53	0.086	2.24	1.08, 5.37	0.045
IL-4 (log)	80	2.74	0.57, 17.0	0.2	2.68	0.37, 24.0	0.3
IL-8 (log)	80	0.63	0.34, 1.10	0.11	0.38	0.13, 0.94	0.050
IL-18 (log)	80	1.89	0.72, 5.18	0.2	4.85	0.91, 32.7	0.079

Abbreviations: CI = Confidence Interval, OR = Odds Ratio.

## Data Availability

The data supporting the findings of this study are available from the corresponding author upon reasonable request. Data are not publicly available due to privacy and ethical restrictions.

## Appendix A

**Table A1:** Clinical, functional and biochemical characteristics of patients with CKD stages 2–4 stratified by eGFR category.

Variable	CKD2 N = 14 ^1^	CKD3a N = 27 ^1^	CKD3б N = 31 ^1^	CKD4 N = 8 ^1^	*p*-Value ^2^	Overall N = 80 ^1^
Sex					0.2	
Female	5 (36%)	10 (37%)	17 (55%)	6 (75%)		38 (48%)
Male	9 (64%)	17 (63%)	14 (45%)	2 (25%)		42 (53%)
NYHA functional class					0.005	
Class I	0 (0%)	1 (3.7%)	0 (0%)	0 (0%)		1 (1.3%)
Class II	2 (14%)	2 (7.4%)	5 (16%)	0 (0%)		9 (11%)
Class III	10 (71%)	8 (30%)	22 (71%)	6 (75%)		46 (58%)
Class IV	1 (7.1%)	3 (11%)	0 (0%)	1 (13%)		5 (6.3%)
None	1 (7.1%)	13 (48%)	4 (13%)	1 (13%)		19 (24%)
Hypertension						
yes	14 (100%)	27 (100%)	31 (100%)	8 (100%)		80 (100%)
HF echocardiographic phenotype					0.9	
HFrEF	7 (50%)	14 (52%)	14 (45%)	3 (38%)		38 (48%)
HFpEF	5 (36%)	8 (30%)	8 (26%)	2 (25%)		23 (29%)
HFmrEF	2 (14%)	5 (19%)	9 (29%)	3 (38%)		19 (24%)
Age at inclusion (years)	66.50 ± 8.70	68.85 ± 10.37	73.03 ± 10.51	70.50 ± 8.33	0.2	70.22 ± 10.11
Gait speed (m/s)	0.75 [0.70; 0.84]	1.50 [0.50; 1.50]	0.60 [0.40; 1.25]	0.60 [0.48; 1.18]	0.066	0.80 [0.49; 1.50]
Handgrip strength, left (kg)	28.20 [25.20; 43.00]	35.12 [17.90; 40.86]	28.80 [16.30; 39.74]	23.90 [14.70; 39.40]	0.7	29.14 [17.60; 40.58]
Handgrip strength, right (kg)	32.05 [29.60; 60.00]	38.22 [24.20; 46.60]	28.90 [18.00; 43.40]	25.10 [16.35; 37.50]	0.3	32.75 [21.00; 43.15]
Muscle strength index (%)	35.77 [29.07; 44.78]	41.60 [29.45; 54.00]	32.70 [25.43; 52.60]	32.49 [17.08; 43.46]	0.7	38.66 [25.52; 52.07]
SPPB score	7.00 [6.00; 8.00]	7.23 [6.29; 7.65]	6.00 [4.00; 8.00]	5.00 [3.00; 7.06]	0.13	7.00 [5.00; 7.83]
Total body water (L)	47.75 [43.70; 55.30]	46.66 [40.50; 48.00]	44.60 [34.30; 47.80]	44.10 [36.55; 55.45]	0.4	46.01 [36.05; 49.41]
Extracellular water (L)	19.15 [16.60; 21.70]	18.31 [16.90; 20.10]	17.50 [13.70; 18.80]	17.95 [14.70; 24.45]	0.3	18.30 [14.80; 20.00]
Intracellular water (L)	28.20 [24.20; 33.70]	28.41 [24.40; 31.10]	27.40 [20.50; 29.41]	25.70 [21.75; 30.80]	0.6	28.15 [21.55; 30.49]
Fat mass (%)	38.80 [25.30; 58.40]	24.50 [18.80; 30.22]	26.28 [15.10; 30.50]	27.90 [17.85; 45.30]	0.056	26.22 [19.45; 35.70]
Skeletal muscle mass (kg)	29.08 ± 9.24	30.22 ± 8.20	26.46 ± 9.52	28.06 ± 10.63	0.5	28.35 ± 9.13
Height (cm)	168.14 ± 9.39	169.25 ± 10.34	165.24 ± 10.30	160.75 ± 9.74	0.14	166.65 ± 10.27
Skeletal muscle mass index (kg/m^2^)	9.85 [8.80; 10.90]	10.12 [9.50; 11.40]	9.96 [7.30; 10.61]	10.10 [8.30; 12.90]	0.5	10.01 [8.25; 11.35]
Body weight (kg)	100.00 [89.00; 130.00]	85.00 [79.00; 94.00]	86.25 [68.00; 91.76]	90.50 [80.15; 103.00]	0.040	87.00 [75.90; 98.50]
Body mass index (kg/m^2^)	36.25 [30.80; 41.40]	32.00 [27.80; 33.20]	30.91 [27.00; 35.10]	34.50 [30.80; 42.90]	0.023	31.90 [28.10; 36.25]
Body surface area (m^2^)	2.12 [1.86; 2.38]	2.00 [1.77; 2.08]	1.88 [1.66; 2.03]	1.94 [1.76; 2.06]	0.2	1.96 [1.76; 2.08]
Lean mass (kg)	65.25 [59.60; 75.60]	63.05 [55.30; 65.60]	61.00 [46.90; 65.60]	60.25 [49.90; 75.70]	0.5	62.92 [49.25; 67.55]
Resistance (50 kHz, Ω)	457.44 ± 100.52	433.99 ± 83.24	473.82 ± 102.73	411.42 ± 109.21	0.4	451.27 ± 97.35
Reactance (50 kHz, Ω)	50.02 [45.74; 56.09]	46.14 [31.16; 50.57]	41.94 [35.86; 51.67]	35.91 [24.50; 56.77]	0.3	44.92 [35.44; 51.91]
eGFR (mL/min/1.73 m^2^)	62.63 ± 11.19	49.75 ± 5.84	39.51 ± 5.32	24.86 ± 4.97	<0.001	45.55 ± 12.66
Calf circumference (cm)	38.21 [35.00; 41.68]	38.50 [37.01; 41.11]	37.82 [33.04; 40.27]	37.00 [36.50; 43.08]	0.4	38.28 [34.77; 41.00]
Mid-arm muscle circumference (cm)	24.01 [23.58; 26.67]	24.28 [22.80; 24.85]	23.54 [22.22; 25.02]	23.36 [22.69; 25.40]	0.7	23.77 [22.79; 25.06]
Mid-arm muscle area (mm^2^)	451.55 [429.97; 565.69]	473.64 [417.51; 487.74]	429.75 [404.70; 493.84]	445.56 [392.59; 513.81]	0.6	452.27 [407.04; 493.76]
Propionic acid (µmol/L)	6325.00 [3440.00; 7554.00]	6490.00 [4560.00; 7609.30]	6280.00 [5070.00; 7108.10]	6626.40 [5329.15; 7451.60]	0.9	6354.35 [4635.00; 7226.60]
Isobutyric acid (µmol/L)	11,703.30 [1440.00; 14,743.00]	12,140.60 [9720.00; 13,352.19]	9961.90 [8510.00; 12,007.90]	14,773.72 [11,087.28; 19,322.47]	0.092	10,950.00 [8540.00; 13,456.10]
Butyric acid (µmol/L)	15,350.00 [10,000.00; 17,900.00]	14,700.00 [10,900.00; 18,700.00]	18,200.00 [10,400.00; 19,800.00]	15,372.50 [10,684.60; 16,753.00]	0.6	15,500.00 [10,300.00; 19,153.05]
2-Methylbutyric acid (µmol/L)	8865.00 [1330.00; 11,900.00]	9400.60 [5010.00; 11,863.50]	7990.06 [4090.00; 9510.00]	10,012.22 [6222.76; 10,429.60]	0.3	8560.00 [4565.00; 10,479.60]
3-Methylbutyric acid (µmol/L)	12,650.00 [285.00; 23,000.00]	16,087.81 [9710.00; 20,200.00]	12,016.04 [8580.00; 15,720.03]	16,960.54 [10,493.27; 17,826.35]	0.3	14,000.00 [8880.00; 18,000.00]
Pentanoic acid (µmol/L)	479.50 [378.00; 574.00]	471.27 [316.00; 628.00]	478.00 [321.00; 604.00]	486.98 [306.00; 633.03]	>0.9	477.39 [335.00; 605.00]
4-Methylpentanoic acid (µmol/L)	161.31 [118.00; 175.12]	169.57 [117.00; 212.00]	152.87 [122.00; 187.71]	164.20 [151.48; 190.55]	0.8	157.15 [124.00; 194.19]
Hexanoic acid (µmol/L)	1603.64 [759.00; 2143.11]	1958.86 [1090.00; 2399.43]	1365.46 [1050.00; 2150.00]	1817.11 [1255.00; 2335.79]	0.6	1565.00 [1085.00; 2277.81]
NT-proBNP (pg/mL)	2421.51 [1924.12; 3761.25]	2061.72 [957.43; 3767.00]	1803.19 [1054.34; 3055.56]	2985.97 [423.33; 5213.72]	0.7	2108.40 [1108.80; 3684.54]
IL-4 (pg/mL)	0.21 [0.08; 0.41]	0.19 [0.00; 0.47]	0.08 [0.00; 0.39]	0.00 [0.00; 0.47]	0.5	0.13 [0.00; 0.40]
IL-6 (pg/mL)	9.18 [6.96; 11.21]	9.72 [3.68; 19.47]	10.54 [6.28; 20.65]	15.14 [5.42; 26.79]	0.8	10.00 [5.56; 18.47]
IL-8 (pg/mL)	30.65 [20.91; 35.76]	12.29 [8.08; 22.33]	22.65 [13.38; 45.44]	18.07 [10.46; 40.16]	0.078	21.11 [9.88; 39.33]
IL-10 (pg/mL)	4.40 [0.00; 6.77]	4.80 [2.40; 8.71]	4.80 [3.60; 7.82]	7.03 [4.99; 8.71]	0.5	4.90 [3.20; 8.00]
IL-18 (pg/mL)	239.56 [181.82; 291.34]	305.22 [201.07; 418.57]	250.49 [190.86; 420.21]	302.37 [248.45; 438.18]	0.3	274.79 [197.08; 403.97]
Creatinine (µmol/L)	98.00 [84.00; 107.00]	124.38 [106.00; 129.50]	126.12 [114.00; 138.00]	152.50 [143.70; 246.00]	<0.001	124.19 [106.50; 137.08]
Urea (mmol/L)	7.31 [4.90; 7.92]	8.05 [7.05; 10.80]	8.04 [6.80; 9.00]	14.57 [11.40; 18.46]	<0.001	8.04 [7.01; 10.54]
LVEF (%)	41.80 [34.00; 50.00]	40.35 [30.00; 45.68]	42.00 [28.00; 52.00]	46.50 [27.50; 53.50]	0.9	41.18 [30.00; 50.00]
LV mass index (g/m^2^)	107.43 [88.64; 118.35]	89.64 [4.88; 111.36]	111.95 [98.47; 133.61]	107.02 [39.92; 130.79]	0.023	104.89 [82.89; 125.69]
IVS thickness (cm)	1.20 [1.10; 1.25]	1.18 [1.13; 1.21]	1.20 [1.10; 1.20]	1.17 [1.10; 1.23]	>0.9	1.20 [1.10; 1.21]
Posterior wall thickness (cm)	1.18 [1.00; 1.21]	1.12 [1.09; 1.16]	1.10 [1.00; 1.20]	1.19 [1.10; 1.22]	0.6	1.12 [1.09; 1.20]
LV EDV (mL)	128.64 [108.00; 142.55]	125.94 [97.00; 159.18]	108.00 [88.00; 167.00]	131.25 [106.50; 160.00]	0.6	125.47 [97.00; 159.09]
LV ESV (mL)	76.34 [53.05; 93.39]	79.55 [55.00; 93.73]	64.32 [46.00; 103.12]	86.07 [49.50; 105.00]	0.6	76.34 [52.00; 99.00]
LV EDD (cm)	5.35 [5.00; 5.50]	5.32 [4.98; 5.80]	5.00 [4.60; 5.80]	5.50 [4.85; 5.61]	0.7	5.31 [4.80; 5.70]
Sex (BIA)	9.00 (64.29%)	17.00 (62.96%)	14.00 (45.16%)	2.00 (25.00%)	0.2	42.00 (52.50%)
Appendicular lean mass (kg)	19.03 [17.85; 20.57]	16.89 [15.16; 18.98]	16.37 [15.12; 18.44]	16.86 [15.75; 17.53]	0.036	16.88 [15.70; 18.86]
Appendicular skeletal muscle mass (kg/m^2^)	6.77 [6.21; 7.31]	6.00 [5.75; 6.26]	6.13 [5.68; 6.40]	6.34 [5.89; 7.11]	0.004	6.10 [5.73; 6.58]
Maximal handgrip strength (kg)	32.05 [29.60; 60.00]	38.50 [24.20; 46.60]	30.00 [18.00; 43.40]	25.55 [17.05; 42.00]	0.5	32.90 [21.25; 43.68]
Sarcopenia					0.3	
no	9 (64%)	10 (37%)	17 (55%)	5 (63%)		41 (51%)
sarcopenia	5 (36%)	17 (63%)	14 (45%)	3 (38%)		39 (49%)

^1^ n (%); Mean ± SD; Median [Q1; Q3]. ^2^ Fisher’s exact test; Kruskal–Wallis rank sum test.

**Table A2:** Characteristics of patients according to echocardiographic heart failure phenotype.

Variable	HFrEF N = 38 ^1^	HFpEF N = 23 ^1^	HfmrEF N = 19 ^1^	*p*-Value ^2^	Overall N = 80 ^1^
Sex				0.007	
Female	11 (29%)	15 (65%)	12 (63%)		38 (48%)
Male	27 (71%)	8 (35%)	7 (37%)		42 (53%)
NYHA functional class				0.072	
Class I	0 (0%)	1 (4.3%)	0 (0%)		1 (1.3%)
Class II	3 (7.9%)	3 (13%)	3 (16%)		9 (11%)
Class III	18 (47%)	13 (57%)	15 (79%)		46 (58%)
Class IV	3 (7.9%)	2 (8.7%)	0 (0%)		5 (6.3%)
None	14 (37%)	4 (17%)	1 (5.3%)		19 (24%)
Hypertension					
yes	38 (100%)	23 (100%)	19 (100%)		80 (100%)
Chronic kidney disease stage				0.9	
CKD 2	7 (18%)	5 (22%)	2 (11%)		14 (18%)
CKD 3a	14 (37%)	8 (35%)	5 (26%)		27 (34%)
CKD 3б	14 (37%)	8 (35%)	9 (47%)		31 (39%)
CKD 4	3 (7.9%)	2 (8.7%)	3 (16%)		8 (10%)
Age at inclusion (years)	66.84 ± 9.24	70.52 ± 8.74	76.63 ± 10.58	0.001	70.22 ± 10.11
Gait speed (m/s)	1.38 [0.60; 1.50]	0.80 [0.46; 1.30]	0.50 [0.23; 0.80]	<0.001	0.80 [0.49; 1.50]
Handgrip strength, left (kg)	37.03 [28.60; 42.18]	25.57 [15.50; 39.00]	18.40 [10.30; 30.20]	0.002	29.14 [17.60; 40.58]
Handgrip strength, right (kg)	39.45 [30.60; 44.40]	28.90 [18.00; 41.00]	23.60 [16.10; 40.26]	0.008	32.75 [21.00; 43.15]
Muscle strength index (%)	41.46 [29.45; 52.89]	35.48 [24.25; 44.79]	29.87 [18.51; 47.43]	0.2	38.66 [25.52; 52.07]
SPPB score	7.00 [6.00; 7.65]	7.15 [4.00; 9.00]	5.00 [3.00; 7.00]	0.006	7.00 [5.00; 7.83]
Total body water (L)	47.23 [46.00; 54.40]	37.60 [32.80; 48.90]	38.80 [33.80; 46.80]	<0.001	46.01 [36.05; 49.41]
Extracellular water (L)	19.02 [18.02; 21.70]	15.00 [13.60; 19.00]	16.70 [13.50; 18.40]	<0.001	18.30 [14.80; 20.00]
Intracellular water (L)	29.10 [27.80; 32.90]	22.30 [19.00; 29.10]	22.10 [20.50; 29.10]	<0.001	28.15 [21.55; 30.49]
Fat mass (%)	26.03 [22.30; 33.20]	24.00 [20.10; 37.90]	28.50 [13.20; 39.50]	0.9	26.22 [19.45; 35.70]
Skeletal muscle mass (kg)	32.79 ± 7.75	24.38 ± 8.00	24.27 ± 9.20	<0.001	28.35 ± 9.13
Height (cm)	171.89 ± 8.00	161.97 ± 8.66	161.84 ± 11.35	<0.001	166.65 ± 10.27
Skeletal muscle mass index (kg/m^2^)	10.25 [9.90; 12.00]	9.30 [7.00; 10.80]	8.60 [7.20; 10.10]	0.001	10.01 [8.25; 11.35]
Body weight (kg)	90.16 [85.00; 107.00]	84.00 [66.00; 93.20]	80.00 [74.00; 91.50]	0.031	87.00 [75.90; 98.50]
Body mass index (kg/m^2^)	31.85 [29.32; 36.10]	31.80 [27.10; 36.40]	32.50 [23.30; 39.20]	0.8	31.90 [28.10; 36.25]
Body surface area (m^2^)	2.03 [1.96; 2.19]	1.75 [1.63; 2.06]	1.82 [1.73; 1.95]	<0.001	1.96 [1.76; 2.08]
Lean mass (kg)	65.13 [62.70; 74.40]	51.40 [44.80; 66.80]	53.00 [46.20; 63.90]	<0.001	62.92 [49.25; 67.55]
Resistance (50 kHz, Ω)	419.55 ± 81.53	479.79 ± 93.48	480.18 ± 114.90	0.014	451.27 ± 97.35
Reactance (50 kHz, Ω)	44.69 [31.55; 48.36]	49.03 [38.53; 60.17]	42.94 [35.47; 53.75]	0.12	44.92 [35.44; 51.91]
eGFR (mL/min/1.73 m^2^)	46.49 ± 12.47	46.95 ± 13.20	41.96 ± 12.35	0.3	45.55 ± 12.66
Calf circumference (cm)	38.99 [37.01; 41.51]	35.00 [33.04; 39.00]	38.00 [33.97; 41.14]	0.012	38.28 [34.77; 41.00]
Mid-arm muscle circumference (cm)	24.82 [23.75; 26.72]	23.54 [22.22; 24.58]	23.01 [21.51; 23.72]	<0.001	23.77 [22.79; 25.06]
Mid-arm muscle area (mm^2^)	478.85 [447.96; 566.20]	429.97 [398.72; 473.20]	415.64 [354.01; 452.31]	<0.001	452.27 [407.04; 493.76]
Propionic acid (µmol/L)	6560.00 [4640.00; 7800.00]	6028.30 [4560.00; 6630.00]	6612.80 [4630.00; 7554.00]	0.3	6354.35 [4635.00; 7226.60]
Isobutyric acid (µmol/L)	10,950.00 [6710.00; 13,040.54]	10,001.10 [8510.00; 12,406.60]	13,300.00 [10,500.00; 15,200.00]	0.063	10,950.00 [8540.00; 13,456.10]
Butyric acid (µmol/L)	16,250.00 [10,500.00; 18,600.00]	15,358.50 [10,200.00; 19,700.00]	13,759.20 [10,100.00; 21,700.00]	>0.9	15,500.00 [10,300.00; 19,153.05]
2-Methylbutyric acid (µmol/L)	8700.00 [5010.00; 10,700.00]	7190.00 [3470.00; 10,459.20]	9510.00 [7250.00; 10,400.00]	0.3	8560.00 [4565.00; 10,479.60]
3-Methylbutyric acid (µmol/L)	14,600.00 [8580.00; 18,000.00]	10,986.53 [7030.00; 17,652.69]	16,377.60 [11,600.00; 18,200.00]	0.3	14,000.00 [8880.00; 18,000.00]
Pentanoic acid (µmol/L)	533.62 [365.00; 657.00]	417.00 [316.00; 495.00]	479.00 [249.00; 614.07]	0.094	477.39 [335.00; 605.00]
4-Methylpentanoic acid (µmol/L)	152.44 [122.00; 179.99]	172.00 [143.00; 266.29]	157.31 [115.00; 195.29]	0.3	157.15 [124.00; 194.19]
Hexanoic acid (µmol/L)	1845.00 [1100.00; 2400.55]	1196.13 [718.00; 2063.10]	1570.00 [1210.00; 2049.90]	0.2	1565.00 [1085.00; 2277.81]
NT-proBNP (pg/mL)	2504.66 [1389.92; 4826.68]	2051.00 [1163.27; 2499.08]	1607.77 [684.96; 3607.83]	0.092	2108.40 [1108.80; 3684.54]
IL-4 (pg/mL)	0.13 [0.00; 0.41]	0.08 [0.00; 0.27]	0.18 [0.00; 0.62]	0.5	0.13 [0.00; 0.40]
IL-6 (pg/mL)	11.28 [7.23; 19.47]	6.64 [3.46; 12.40]	14.38 [4.20; 23.10]	0.041	10.00 [5.56; 18.47]
IL-8 (pg/mL)	23.07 [9.54; 41.01]	15.39 [8.67; 39.17]	19.31 [12.29; 43.78]	0.8	21.11 [9.88; 39.33]
IL-10 (pg/mL)	5.71 [3.20; 8.71]	3.80 [2.20; 7.59]	6.77 [4.00; 9.24]	0.13	4.90 [3.20; 8.00]
IL-18 (pg/mL)	269.59 [196.98; 418.57]	244.57 [172.88; 320.75]	325.40 [231.29; 450.23]	0.2	274.79 [197.08; 403.97]
Creatinine (µmol/L)	128.00 [123.00; 137.16]	112.00 [87.00; 129.50]	119.00 [107.00; 138.00]	0.022	124.19 [106.50; 137.08]
Urea (mmol/L)	8.07 [7.19; 10.80]	7.70 [6.65; 9.86]	8.40 [5.20; 11.06]	0.4	8.04 [7.01; 10.54]
LVEF (%)	29.00 [25.00; 35.00]	52.00 [52.00; 52.00]	47.00 [42.38; 48.00]	<0.001	41.18 [30.00; 50.00]
LV mass index (g/m^2^)	106.89 [70.90; 126.06]	96.70 [82.17; 118.35]	105.36 [92.85; 124.80]	0.8	104.89 [82.89; 125.69]
IVS thickness (cm)	1.19 [1.10; 1.21]	1.20 [1.10; 1.24]	1.20 [1.10; 1.21]	0.8	1.20 [1.10; 1.21]
Posterior wall thickness (cm)	1.10 [1.09; 1.20]	1.15 [1.00; 1.21]	1.12 [1.10; 1.20]	0.8	1.12 [1.09; 1.20]
LV EDV (mL)	157.38 [127.27; 194.00]	97.00 [88.00; 108.00]	103.39 [92.00; 130.00]	<0.001	125.47 [97.00; 159.09]
LV ESV (mL)	94.37 [80.99; 143.00]	47.00 [42.00; 57.61]	54.91 [50.00; 65.00]	<0.001	76.34 [52.00; 99.00]
LV EDD (cm)	5.55 [5.36; 6.20]	4.80 [4.50; 5.00]	5.00 [4.60; 5.40]	<0.001	5.31 [4.80; 5.70]
Sex (BIA)	27.00 (71.05%)	8.00 (34.78%)	7.00 (36.84%)	0.007	42.00 (52.50%)
Appendicular lean mass (kg)	17.98 [16.46; 19.85]	16.27 [14.55; 18.57]	16.00 [14.98; 17.19]	0.009	16.88 [15.70; 18.86]
Appendicular skeletal muscle mass (kg/m^2^)	6.06 [5.71; 6.43]	6.21 [5.87; 6.74]	6.19 [5.57; 6.93]	0.5	6.10 [5.73; 6.58]
Maximal handgrip strength (kg)	40.72 [31.10; 46.95]	28.90 [18.00; 41.00]	23.60 [16.60; 40.26]	0.006	32.90 [21.25; 43.68]
Sarcopenia				0.2	
no	16 (42%)	15 (65%)	10 (53%)		41 (51%)
sarcopenia	22 (58%)	8 (35%)	9 (47%)		39 (49%)

^1^ n (%); Mean ± SD; Median [Q1; Q3]. ^2^ Pearson’s Chi-squared test; Fisher’s exact test; Kruskal–Wallis rank sum test.
